# Value of ^18^F-FDG PET/MRI in the Preoperative Assessment of Resectable Esophageal Squamous Cell Carcinoma: A Comparison With ^18^F-FDG PET/CT, MRI, and Contrast-Enhanced CT

**DOI:** 10.3389/fonc.2022.844702

**Published:** 2022-02-28

**Authors:** Fei Wang, Rui Guo, Yan Zhang, Boqi Yu, Xiangxi Meng, Hanjing Kong, Yang Yang, Zhi Yang, Nan Li

**Affiliations:** ^1^ Key Laboratory of Carcinogenesis and Translational Research (Ministry of Education/Beijing), NMPA Key Laboratory for Research and Evaluation of Radiopharmaceuticals (National Medical Products Administration), Department of Nuclear Medicine, Peking University Cancer Hospital & Institute, Beijing, China; ^2^ Beijing United Imaging Research Institute of Intelligent Imaging, UIH Group, Beijing, China

**Keywords:** PET/MRI, PET/CT, MRI, esophageal cancer, staging

## Abstract

**Objectives:**

To investigate the value of ^18^F-FDG PET/MRI in the preoperative assessment of esophageal squamous cell carcinoma (ESCC) and compare it with ^18^F-FDG PET/CT, MRI, and CECT.

**Methods:**

Thirty-five patients with resectable ESCC were prospectively enrolled and underwent PET/MRI, PET/CT, and CECT before surgery. The primary tumor and regional lymph nodes were assessed by PET/MRI, PET/CT, MRI, and CECT, respectively, and the diagnostic efficiencies were determined with postoperative pathology as a reference standard. The predictive role of imaging and clinical parameters on pathological staging was analyzed.

**Results:**

For primary tumor staging, the accuracy of PET/MRI, MRI, and CECT was 85.7%, 77.1%, and 51.4%, respectively. For lymph node assessment, the accuracy of PET/MRI, PET/CT, MRI, and CECT was 96.2%, 92.0%, 86.8%, and 86.3%, respectively, and the AUCs were 0.883, 0.745, 0.697, and 0.580, respectively. PET/MRI diagnosed 13, 7, and 6 more stations of lymph node metastases than CECT, MRI, and PET/CT, respectively. There was a significant difference in SUV_max_, TLG, and tumor wall thickness between T1-2 and T3 tumors (*p* = 0.004, 0.024, and < 0.001, respectively). Multivariate analysis showed that thicker tumor wall thickness was a predictor of a higher T stage (*p* = 0.040, OR = 1.6).

**Conclusions:**

^18^F-FDG PET/MRI has advantages over ^18^F-FDG PET/CT, MRI, and CECT in the preoperative assessment of primary tumors and regional lymph nodes of ESCC. ^18^F-FDG PET/MRI may be a potential supplement or alternative imaging method for preoperative staging of ESCC.

## Introduction

Esophageal cancer is the seventh most prevalent malignancy worldwide, with the sixth leading cause of cancer-related mortality (1). Accurate staging is essential for treatment selection and prognosis prediction for patients with esophageal cancer. Imaging plays a critical role in tumor staging. Currently, the commonly used imaging methods for staging include computed tomography (CT), endoscopic ultrasonography (EUS), and positron emission tomography (PET)/CT. However, accurate preoperative staging remains a challenge (2).

In the description of the primary tumor (T staging) of esophageal cancer, it is difficult to distinguish the layers of the esophageal wall on CT due to the poor contrast of soft tissue. The application of CT is limited to distinguishing T3 and T4 tumors in the T staging of esophageal cancer (3). In the assessment of regional lymph nodes (N staging), the determination only depends on the size of the lymph nodes by CT, with low accuracy. EUS or EUS combined with fine-needle aspiration biopsy (FNAB) reveals high accuracy in T and N staging, but its application is limited by operator dependency, risk of hemorrhage (0.13% morbidity rate), and perforation (0.03%-0.07% morbidity rate), inability to pass through the stenosis (20%-30% morbidity rate), and the scope of the examination (4, 5). In clinical practice, ^18^F-fluorodeoxyglucose (^18^F-FDG) PET/CT has limitations in T staging of esophageal cancer due to its resolution but shows high specificity in N staging, with poor sensitivity and some false-positive results. With superior soft-tissue contrast, magnetic resonance imaging (MRI) may distinguish the layers of the esophageal wall and adjacent lymph nodes and has the features of multi-parametric and functional imaging. MRI revealed better accuracy in T staging and higher sensitivity in N staging than CT, but there were still some primary tumors and lymph nodes that were difficult to detect and accurately describe by MRI. Therefore, a more accurate and reliable noninvasive preoperative staging method is desired.

^18^F-FDG PET/MRI provides both metabolic and anatomical information about the tumor and combines the advantages of MRI’s superior soft-tissue resolution and multi-parametric imaging, which can detect more malignant lesions than PET/CT, leading to changes in TNM staging (6, 7). Previous studies revealed ^18^F-FDG PET/MRI to be superior to PET/CT in T staging and at least comparable to PET/CT in N and M staging of a variety of tumors (8, 9). In addition, imaging parameters such as the standardized uptake value (SUV) and apparent diffusion coefficient (ADC) of tumors may correlate with staging and prognosis (10). Preliminary studies showed that PET/MRI may overcome the inherent limitations of PET/CT and CT in T staging of esophageal cancer, and has advantages over other imaging methods in N staging (11). However, there are few studies of PET/MRI in the assessment of esophageal cancer, and the value needs to be further explored. Therefore, the purpose of our study was to compare the diagnostic efficiency of PET/MRI, PET/CT, MRI, and contrast-enhanced CT (CECT) in the preoperative assessment of primary tumors and regional lymph nodes of esophageal cancer and to explore the role of imaging and clinical parameters in predicting pathological stages.

## Materials And Methods

### Patient Enrollment

The study was approved by the Ethics Committee of the Peking University Cancer Hospital & Institute (No.2018KT110-GZ01) and informed consent was obtained from all individual participants included in the study. From September 2019 to April 2021, 35 patients with biopsy-confirmed and untreated resectable esophageal squamous cell carcinoma (ESCC) were prospectively enrolled in this study. Exclusion criteria were unwillingness to undergo surgical resection, pregnancy, history of other malignant tumors, intolerance of long-term supine, cognitive or language impairment, contraindications for MRI examination (e.g., claustrophobia, metal implants or electronic devices, etc.), or diabetes with uncontrollable blood glucose higher than 10.0 mmol/L. ^18^F-FDG PET/MRI, ^18^F-FDG PET/CT, and CECT were performed within two weeks before surgery. The clinicopathological characteristics of the patients are shown in **Table 1**.

**Table 1:** Clinicopathological Characteristics.

Characteristic	Data	Percentage
**Total**	35	100.0%
**Age**	62 ± 7	
**Gender**		
Male	28	80.0%
Female	7	20.0%
**Location**		
Upper	3	8.6%
Middle	13	37.1%
Lower	19	54.3%
**Histologic differentiation**		
Well-moderately differentiated	22	62.9%
Poorly differentiated	13	37.1%
**T Stage**		
T1	15	42.9%
T2	9	25.7%
T3	11	31.4%
**N Stage**		
N0	20	57.1%
N+	15	42.9%

### Image Acquisition

#### PET/CT Image Acquisition

PET/CT was performed after fasting for at least six hours, with patients’ blood glucose lower than 10.0 mmol/L. The acquisition was performed approximately 60 ± 10 minutes after an injection of a weight-adapted activity of ^18^F-FDG (3.7 MBq/kg) with a hybrid scanner (Biograph mCT, Siemens, Erlangen, Germany). The scan ranged from the skull base to the upper thighs. Attenuation correction was performed using low-dose CT without a contrast agent. PET scan was acquired with a speed of 1.0 mm/s. The ordered-subsets expectation maximization (OSEM) method was used for PET image reconstruction.

#### PET/MR Image Acquisition

PET/MR images were obtained immediately after PET/CT scan without additional ^18^F-FDG injection using an integrated PET/MRI system (uPMR 790, United Imaging Healthcare, Shanghai, China) with a 12-channel body coil, combining a time-of-flight PET scanner and 3.0T MR. PET and MR images were acquired simultaneously. The scan ranged from the lower neck to the upper abdomen. For attenuation correction, a respiratory-triggered T1-weighted sequence with the Dixon technique was acquired. Diagnostic MR imaging consisted of axial and sagittal respiratory-triggered T2-weighted imaging (T2WI), axial respiratory-triggered T2 high-resolution imaging of the primary tumor with small-field of view (FOV) imaging technology, and diffusion-weighted imaging (DWI) with b-values of 50 s/mm^2^ and 800 s/mm^2^. No intravenous contrast agent was used. The mean acquisition time of PET/MRI was approximately 30-40 minutes.

### Image Analysis

Images were displayed on the workstation provided by the vendor and reviewed by two experienced physicians who were blinded to the pathological results. In case of disagreement, they decided through discussion. T-staging was assigned based on the depth of tumor invasion and the relationship with surrounding fat and structure. The criteria for preoperative T staging by PET/MRI and MRI were T0, with an uptake no higher than that of the surrounding esophagus, and no intensity change; T1, with interrupted medium to high intensity in mucosa and submucosa and intact low intensity in the muscle layer, with an uptake higher than that of the surrounding esophagus; T2, with interrupted low intensity in the muscle layer and intact high intensity in the adventitia, with an uptake higher than that of the surrounding esophagus; T3, with interrupted high intensity in the adventitia and with a fat gap between the lesion and adjacent structures, with an uptake higher than that of the surrounding esophagus; T4, the fat gap between the lesion and adjacent structures disappeared, with an uptake higher than that of the surrounding esophagus (12). The preoperative T staging criteria of CECT were as follows: T0, with no change in density or thickness; T1, with low density of lesions relative to normal mucosa and submucosa; T2, the esophageal wall was thickened, the outer edge was smooth, and the fat surface around the lesion was clear; T3, the esophageal wall was thickened, the outer edge was irregular, and the surrounding fat surface was unclear; T4, the fat gap between esophageal lesions and adjacent structures disappeared. It was difficult for PET/CT to provide information on esophageal wall layers, so PET/CT was excluded from the T-staging comparison.

For PET/MRI, PET/CT, and MRI, lymph nodes with uptake above the level of the mediastinum background or with a disappearance of fatty hilum or with eccentric cortical thickening were considered metastases, regardless of size. For CECT, lymph nodes with a short-axis diameter of more than 5 mm in the supraclavicular station, or with a short-axis diameter of more than 10 mm in other stations were considered metastases (13). Lymph nodes with an uptake equal to or lower than the level of the mediastinum background, symmetric uptake in bilateral hilar, target-ring, pure high-density, or with calcification were judged as benign, regardless of size. The diagnostic performance of PET/MRI, PET/CT, MRI, and CECT was determined with postoperative pathology as a reference standard. All suspected positive lymph nodes were surgically removed, and all surgically removed lymph nodes were analyzed.

The imaging parameters analyzed included maximum standardized uptake value (SUV_max_), metabolic volume (MTV), total glucose glycolysis (TLG), minimum and mean value of ADC (ADC_min_, ADC_mean_), tumor wall thickness, measured by PET/MRI, the difference in CT values between plain and enhanced CT (△HU), and the maximum short-axis diameter (D_max_) of lymph nodes, measured by CECT.

### Statistical Analysis

Continuous variables are presented as the mean ± standard deviation (SD), and classified variables are presented as frequencies and percentages. SPSS software (version 22.0, IBM Corp.) and MedCalc software (version 19.0.4, MedCalc Software Ltd.) were used for statistical analysis. Comparisons of PET/MRI, PET/CT, MRI, and CECT in lymph node assessment were performed using the McNemar test, Pearson chi-square test, or Fisher’s exact test, and the diagnostic efficiency of the four methods was assessed using the receiver operating characteristic (ROC) curve. An independent sample t-test was used to test the difference in imaging and clinical parameters between tumors with different T and N stages tumors. Logistic regression analysis was performed for multivariate analysis. P values less than 0.05 were considered statistically significant.

## Results

### Primary Tumor Assessment

Postoperative pathology confirmed that there were 15 cases of T1 disease, 9 cases of T2 disease, and 11 cases of T3 disease. The mean SUV_max_ of the primary tumors was 9.7 ± 5.8 (1.7 - 20.8), among which, the mean SUV_max_ was 4.7 ± 3.0 (1.7 - 10.7) for T1 tumors, 13.0 ± 5.3 (3.1 - 19.4) for T2 tumors, and 13.7 ± 3.9 (8.2 - 20.8) for T3 tumors. There was a significant difference in SUV_max_ between T1 and T2 tumors (*p* < 0.001) but no significant difference in SUV_max_ between T2 and T3 tumors (*p* = 0.709).

**Figure 1:**
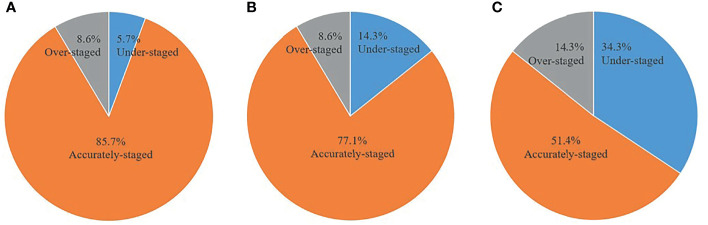
Comparison of accuracy between PET/MRI, MRI, and CECT in primary tumor assessment. **(A)** PET/MRI. **(B)** MRI. **(C)** CECT.

**Figure 2:**
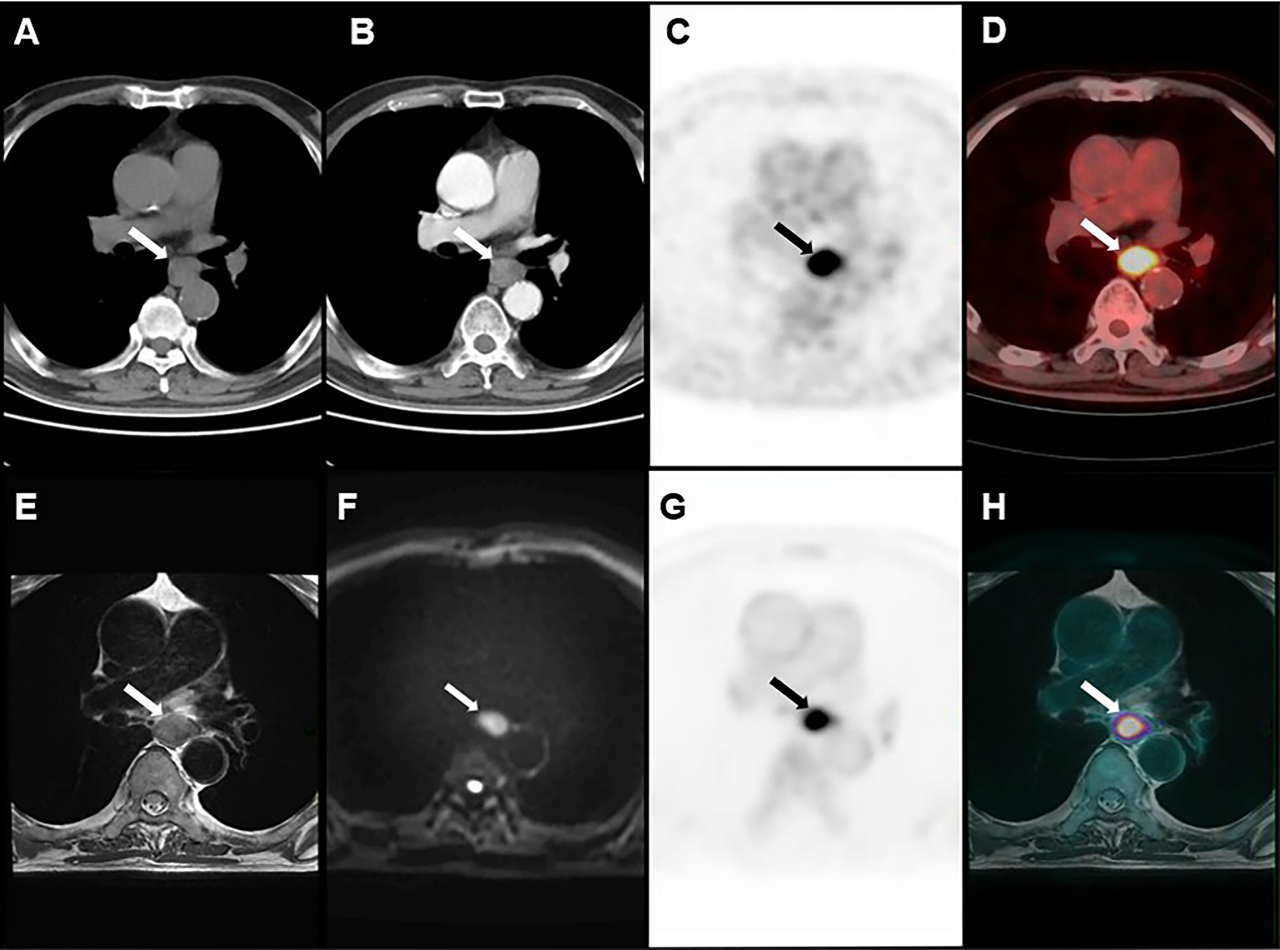
Images of a 72-year-old man with ESCC. **(A)** plain CT. **(B)** CECT. **(C, D)** PET/CT. **(E–H)** PET/MRI. T3 disease was considered by PET/MRI, which was consistent with postoperative pathology.

The accuracy of distinguishing T1, T2, and T3 tumors was 86.7%, 77.8%, and 90.9% for PET/MRI, respectively; 66.7%, 77.8%, and 90.9% for MRI, respectively; and 40.0%, 44.4%, and 72.7% for CECT, respectively (**Table 2**). Thirty (85.7%) primary tumors were accurately staged by PET/MRI, 27 (77.1%) by MRI, and 18 (51.4%) by CECT. Three cases were over-staged, and 2 cases were under-staged by PET/MRI (**Figure 1A**). Meanwhile, 3 cases were over-staged and 5 cases were under-staged by MRI (**Figure 1B**), 5 cases were over-staged and 12 cases were under-staged by CECT (**Figure 1C**). A typical case is shown in **Figure 2**.

**Table 2:** Comparison of primary tumor assessment between PET/MRI, MRI, and CECT.

Pathological stage	PET/MRI				Accuracy	MRI				Accuracy	CECT				Accuracy
	T0	T1	T2	T3		T0	T1	T2	T3		T0	T1	T2	T3	
T1 (n = 15)	1	13	1	0	86.7%	4	10	1	0	66.7%	7	6	2	0	40.0%
T2 (n = 9)	0	0	7	2	77.8%	0	0	7	2	77.8%	1	1	4	3	44.4%
T3 (n = 11)	0	0	1	10	90.9%	0	0	1	10	90.9%	0	0	3	8	72.7%
Accurately staged	0	13	7	10	85.7%	0	10	7	10	77.1%	0	6	4	8	51.4%

### Regional Lymph Node Assessment

A total of 847 lymph nodes (24 ± 9 per patient) from 212 stations were dissected in 35 patients, including the lymph nodes from the lower neck to the upper abdomen. Among those, there were 32 (23 stations) metastatic lymph nodes and 815 (189 stations) non-metastatic lymph nodes. The mean SUV_max_ of metastatic and non-metastatic lymph nodes was 2.1 ± 1.6 and 1.3 ± 1.1, respectively, and the mean D_max_ was 5.8 ± 2.0 mm and 4.4 ± 1.6 mm, respectively. There were significant differences in SUV_max_ and D_max_ between metastatic and non-metastatic lymph nodes (*p* = 0.001 and *p* < 0.001).

#### Total Analysis

**Figure 3:**
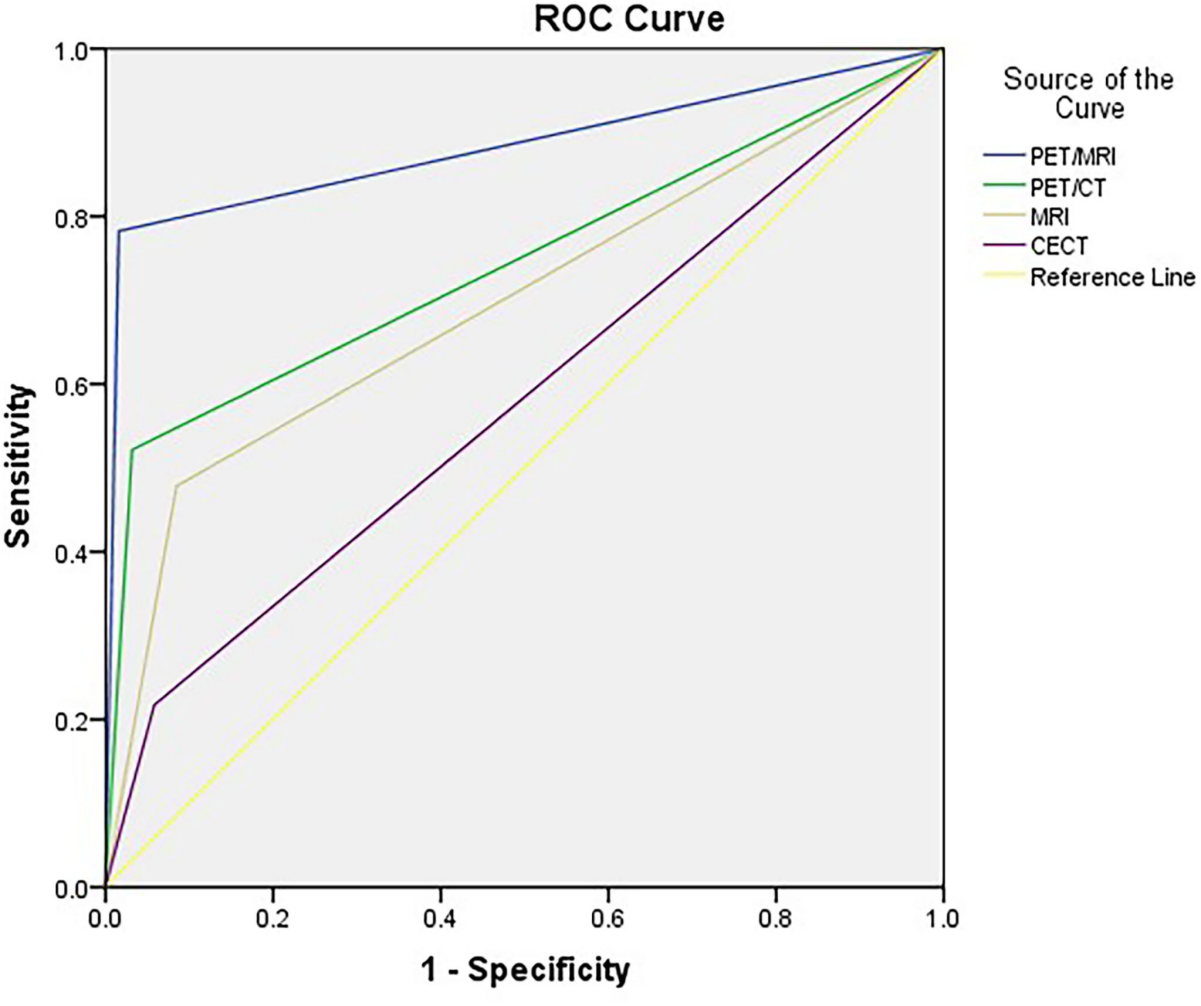
ROC curves for PET/MRI, PET/CT, MRI, and CECT in lymph node assessment. AUCs were 0.883, 0.745, 0.697, and 0.580 for PET/MRI, PET/CT, MRI, and CECT, respectively.

**Figure 4:**
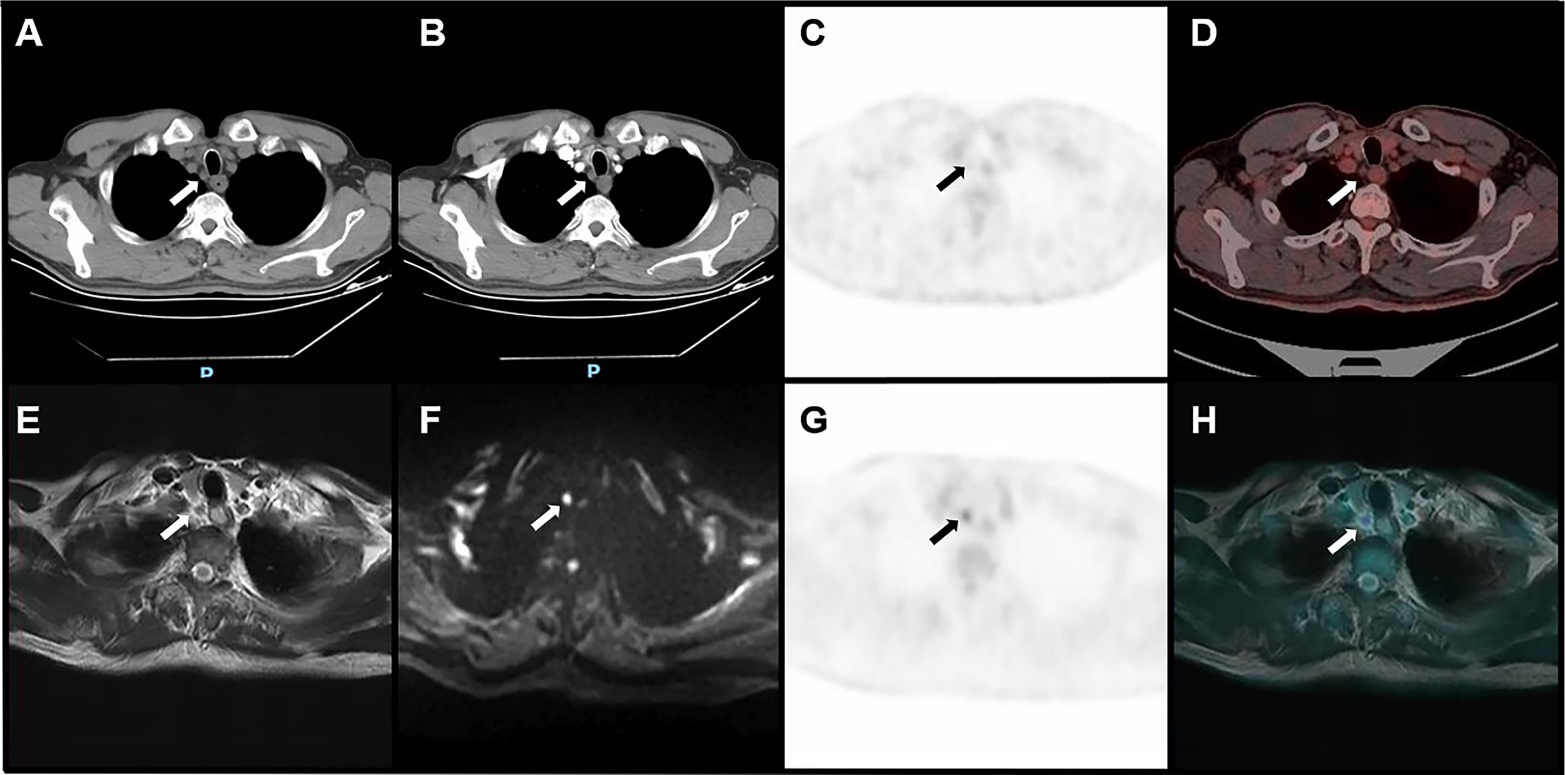
Image of a right upper paratracheal metastatic lymph node confirmed by pathology in a 66-year-old male with ESCC. CT **(A)**, plain CT; **(B)**, CECT showed that the short diameter of lymph nodes was 5 mm. PET/CT **(C, D)** showed that the uptake of the lymph node was equal to the level of the mediastinum background. Both CECT and PET/CT suggested that the lymph node was non-metastatic. PET/MRI **(E–H)** showed that the uptake of the lymph node was higher than the level of the mediastinum background, with slight hyperintensity on T2WI and hyperintensity on DWI, suggesting metastatic lymph nodes.

The accuracy of PET/MRI, PET/CT, MRI, and CECT in diagnosing lymph node metastasis was 96.2%, 92.0%, 86.8%, and 86.3%, respectively. The area under the curve (AUC) was 0.883, 0.745, 0.697, and 0.580 for PET/MRI, PET/CT, MRI, and CECT, respectively. Compared with CECT, PET/MRI diagnosed more lymph node metastasis at 13 stations and excluded metastasis at 8 stations. Compared with MRI, PET/MRI diagnosed more lymph node metastasis at 7 stations and excluded metastasis at 13 stations. Compared with PET/CT, PET/MRI diagnosed more lymph node metastasis at 6 stations and excluded metastasis at 3 stations. The diagnostic performances of PET/MRI, PET/CT, MRI, and CECT in lymph node assessment are shown in **Table 3**. The diagnostic efficiencies and differences of PET/MRI, PET/CT, MRI, and CECT in lymph node assessment are shown in **Table 4** and **Figure 3**. A typical case is shown in **Figure 4**.

**Table 3:** Diagnostic performances of PET/MRI, PET/CT, MRI, and CECT in lymph node assessment.

Pathology	Total	PET/MRI		PET/CT		MRI		CECT	
		Positive	Negative	Positive	Negative	Positive	Negative	Positive	Negative
Positive	23	18	5	12	11	11	12	5	18
Negative	189	3	186	6	183	16	173	11	178

**Table 4:** Comparison of lymph node assessment by PET/MRI, PET/CT, MRI, and CECT.

	Group	Sensitivity	Specificity	PPV	NPV	Accuracy	AUC
Efficiency	PET/MRI	78.3%	98.4%	85.7%	97.4%	96.2%	0.883
	PET/CT	52.2%	96.8%	66.7%	94.3%	92.0%	0.745
	MRI	47.8%	91.5%	40.7%	93.5%	86.8%	0.697
	CECT	21.7%	94.2%	31.3%	90.8%	86.3%	0.580
Difference (*p* value)	PET/MRI vs. PET/CT	0.031*	0.250	0.255	0.134	0.044*	0.003*
	PET/MRI vs. MRI	0.016*	< 0.001*	0.002*	0.071	< 0.001*	< 0.001*
	PET/MRI vs. CECT	< 0.001*	0.039*	0.002*	0.006*	< 0.001*	< 0.001*
	PET/CT vs. MRI	1.000	0.021*	0.088	0.739	0.115	0.4183
	PET/CT vs. CECT	0.016*	0.267	0.084	0.186	0.086	< 0.001*
	MRI vs. CECT	0.146	0.405	0.534	0.329	0.887	0.1079

*p < 0.05. PPV, positive predictive value; NPV, negative predictive value; AUC, area under the curve.

#### Subgroup Analysis

Patients were divided into two groups by tumor location, histologic differentiation, and T stage. Subgroup analysis showed that the AUC of PET/MRI in lymph node assessment was superior to PET/CT, MRI, and CECT in each subgroup (**Table 5**). PET/MRI showed more obvious superiority in lymph node assessment in the lower-thoracic group, poorly-differentiated group, and T3 group, which were significantly different from PET/CT, MRI, and CECT.

**Table 5:** Comparison of AUC of PET/MRI, PET/CT, MRI, and CECT in lymph node assessment: subgroup analysis.

		Location		Differentiation		T stage	
	Group	Upper-middle	Lower	Well-moderate	Poor	T1-2	T3
AUC	PET/MRI	0.651	0.925	0.873	0.910	0.834	0.950
	PET/CT	0.516	0.784	0.747	0.737	0.715	0.784
	MRI	0.602	0.729	0.703	0.693	0.641	0.776
	CECT	0.543	0.609	0.556	0.641	0.584	0.576
Difference (*p* value)	PET/MRI vs. PET/CT	0.423	0.005^*^	0.018^*^	0.011^*^	0.050	0.031^*^
	PET/MRI vs. MRI	0.002^*^	< 0.001^*^	0.003^*^	0.042^*^	0.004^*^	0.024^*^
	PET/MRI vs. CECT	0.521	< 0.001^*^	< 0.001^*^	0.017^*^	< 0.001^*^	< 0.001^*^
	PET/CT vs. MRI	0.609	0.383	0.529	0.734	0.410	0.916
	PET/CT vs. CECT	0.092	0.002*	0.002^*^	0.258	0.035^*^	0.012^*^
	MRI vs. CECT	0.725	0.141	0.082	0.739	0.555	0.075

*p < 0.05.

### Univariate and Multivariate Analysis of T or N Staging

There were significant differences in SUV_max_, TLG, and tumor wall thickness between T1-2 and T3 tumors, but no significant differences in MTV, ADC_min_, ADC_mean_, △HU, or any clinical parameters [including sex, age, tobacco and alcohol habits, family history of esophageal cancer, and the serum levels of tumor markers (CA199, CA72.4, CA242, NSE, CYFRA21-1, and SCC)]. No significant differences were observed in any primary tumor imaging parameter or clinical parameter between N0 and N+ patients. The SUV_max_, TLG, and thickness of the tumor were included in the multivariate analysis of T staging, which revealed that thicker tumor wall thickness was a predictor of a higher T stage (T ≥ 3) (*p* = 0.040, OR = 1.6). The results of univariate and multivariate analyses of T or N staging are shown in **Table 6**.

**Table 6:** Univariate and multivariate analysis of T or N staging.

		T stage			Multivariate	N stage		
	Parameters	T1-2	T3	*p* value	*p* value	N0	N+	*p* value
PET/MRI	SUV_max_	7.8 ± 5.6	13.7 ± 3.9	0.004^*^	0.286	10.2 ± 6.1	8.9 ± 5.5	0.525
	MTV(mL)	3.0 ± 2.3	4.5 ± 3.2	0.126	–	3.2 ± 2.2	3.7 ± 3.2	0.589
	TLG	15.3 ± 22.6	34.8 ± 22.8	0.024^*^	0.296	21.2 ± 23.9	21.7 ± 25.3	0.958
	ADC_min_(×10^-3^mm^2^/s)	1.4 ± 0.4	1.2 ± 0.2	0.169	–	1.4 ± 0.4	1.2 ± 0.3	0.156
	ADC_mean_(×10^-3^mm^2^/s)	1.8 ± 0.4	1.6 ± 0.2	0.210	–	1.8 ± 0.4	1.6 ± 0.2	0.305
	Thickness (mm)	7.4 ± 3.2	12.2 ± 3.2	< 0.001^*^	0.040^*^	9.0 ± 3.8	8.8 ± 4.0	0.882
CECT	△HU	34.5 ± 15.4	36.3 ± 14.5	0.755	–	33.5 ± 16.3	37.2 ± 13.2	0.477

*p < 0.05.

## Discussion

The depth of tumor invasion is the key to treatment and surgical options. T1-2 tumors can be treated directly by surgery, while T3-4 tumors often need preoperative neoadjuvant therapy. Previous studies have demonstrated the high accuracy (higher than 80%) of EUS in T staging (14). However, EUS has some limitations, such as operator dependence, inability to pass through the stenoses, and the risk of hemorrhage and perforation, which limit its application. CT, with poor soft-tissue contrast, has limited ability to accurately distinguish T1 and T2 diseases from T3 diseases and is mainly used to distinguish T3 and T4 tumors. Due to the spatial resolution of PET and the low contrast of low-dose CT, PET/CT scans are unable to provide accurate information on esophageal wall stratification and have a limited role in T staging.

MRI, with superior soft-tissue contrast, can display stratification of the esophageal wall and observe the surrounding tissue structure. *In vitro* studies showed that the three layers of the esophageal wall could be distinguished on high-resolution T2WI (15). However, there are still some tumors that cannot be accurately staged by MRI, especially superficial diseases. Previous studies showed that PET/MRI could identify the esophageal wall layer, with acceptable accuracy slightly worse than EUS (11). In this study, we found that PET/MRI could distinguish the stratification of the esophageal wall with better accuracy than CECT in preoperative T staging of ESCC. The diagnostic accuracy of PET/MRI for T1, T2, and T3 diseases (86.7%, 77.8%, and 83.3%, respectively) was superior to that of CECT (40.0%, 44.4%, and 75.0%, respectively). Moreover, PET/MRI may improve the detection of superficial lesions by MRI. Thus, the description of tumor invasion depth may be significantly improved by PET/MRI. Given the poor performance of PET/CT in T staging of esophageal cancer, we believe that PET/MRI may be an effective supplement to PET/CT, MRI, and CECT in T staging of ESCC.

Lymph node staging is a critical prognostic factor for esophageal cancer. Accurate N staging determines the treatment and facilitates complete resection of all positive lymph nodes to improve long-term survival. Conversely, extended lymphadenectomy may increase postoperative complications. At present, the accuracy of common methods is not satisfactory in lymph node assessment. CT only takes the size of lymph nodes as a judgment standard, with low accuracy, as some small lymph nodes are metastatic, whereas inflammatory, reactive, and granulomatous lymph nodes may be enlarged. Previous studies have used different criteria for diagnosing lymph node metastasis, with most studies using a short diameter larger than 10 mm as a criterion (16). However, our study revealed that the short diameter of metastatic lymph nodes in all 23 stations was less than 10 mm, with a mean value of 5.8 ± 2.0 mm. Therefore, it is not accurate to take only the short diameter of lymph nodes as the standard for the diagnosis of metastatic lymph nodes, which has a high false-negative rate.

EUS combined with FNAB revealed high accuracy in the diagnosis of lymph node metastasis, but non-paraesophageal lymph nodes were easily missed due to the limitation of the detection range, and the passage was limited when the lumen was narrow. Furthermore, the lymph nodes near the tumor cannot be punctured. The advantage of PET/CT in N staging of esophageal cancer is of high specificity. However, its sensitivity is low, at only approximately 30%-60%, due to the spatial resolution. Therefore, the detection of small lymph node metastases and the differentiation of paraesophageal lymph nodes from primary tumors remain challenges. Lymph nodes can be detected sensitively on DWI images. Meanwhile, the intensity and internal structure of the lymph nodes can be observed on high-resolution T2WI (17, 18). Therefore, metastatic lymph nodes smaller than 10.0 mm can also be identified by MRI. However, there is an overlap of ADC values or intensity between metastatic and non-metastatic lymph nodes (19). In this condition, the differentiation of benign and malignant lymph nodes is difficult by MRI. PET/MRI may provide additional information for lymph node assessment. Our study found that PET/MRI had better diagnostic efficiency than PET/CT, MRI, and CECT in lymph node assessment of ESCC (AUC: 0.883, 0.745, 0.697, and 0.580, respectively; sensitivity: 78.3%, 52.2%, 47.8%, and 21.7%, respectively). Combining metabolism and morphology, PET/MRI diagnosed 13, 7, and 6 more stations of lymph node metastases than CECT, MRI or PET/CT, respectively, as well as excluded metastases in 8, 13, and 3 stations than CECT, MRI, or PET/CT, respectively. Subgroup analysis in our study also revealed that PET/MRI had more obvious advantages in lower-thoracic, poorly-differentiated, and T3 ESCC. Therefore, we believe that PET/MRI may improve the sensitivity, accuracy, and diagnostic confidence of lymph node assessment, play a complementary or further confirming role and may reduce the risk of biopsy or avoid other additional imaging examinations.

Previous studies have demonstrated that the SUV, MTV, TLG, and ADC may be valuable prognostic factors for esophageal cancer (10, 20). However, whether these parameters can be used for the prediction of the pathological stage is still controversial. For the treatment of esophageal cancer, the muscularis propria is an important dividing line. Our study revealed that SUV_max_, TLG, and tumor wall thickness may be useful for the differentiation of T1-2 and T3 tumors. However, we found that MTV, ADC_min_, ADC_mean,_ △HU, and clinical parameters play a limited role in differentiating T1-2 and T3 tumors, and all the imaging parameters of the primary tumor and clinical parameters play a limited role in differentiating N0 and N+ patients. Therefore, whether these parameters can be used to predict T and N staging remains to be further explored.

Our findings are consistent with those of several previous studies (21), but some studies have found significant differences in ADC and MTV between high and low T-stage tumors (22), and some studies involving PET/CT have reported a significant correlation between tumor SUV_max_ and N stage (23). In the present study, we found that the SUV_max_ and D_max_ of lymph nodes may be useful in differentiating metastatic and non-metastatic lymph nodes. The differences between the results of different studies may be attributed to differences in clinicopathological characteristics or treatment of patients or differences in sample size. In general, the use of imaging parameters to accurately predict the staging of esophageal cancer still requires further study. Furthermore, the application of new techniques may help to improve the efficiency of PET/MRI in predicting T and N staging of esophageal cancer (24–28).

This study has several limitations. First, the limited number of cases included in the analysis may affect the power of the statistical analysis. Second, only a few patients underwent EUS examinations before surgery in our study. Therefore, the diagnostic efficiency of PET/MRI, PET/CT, MRI, and CECT cannot be compared with EUS at the same time. This may be explained by the guidelines published by the National Institute for Health and Care Excellence (NICE) in 2018, which recommend that EUS should be performed only if there is a potential change in treatment after PET/CT (29). Third, since most of our patients were elderly and could not tolerate multiple breath-hold acquisitions, we did not include breath-hold sequences in MRI acquisition, which may improve the observation of lesions. Finally, our patients were surgical patients without distant metastasis; therefore, the value of PET/MRI in M staging was not analyzed.

## Conclusions

In conclusion, ^18^F-FDG PET/MRI has advantages over ^18^F-FDG PET/CT, MRI, and CECT in the preoperative assessment of the primary tumor and regional lymph node of resectable ESCC, especially in the description of the depth of tumor invasion and the sensitivity of lymph node assessment. Furthermore, PET/MRI-derived imaging parameters also contribute to the prediction of T staging and lymph node status. ^18^F-FDG PET/MRI may be a potential supplement or alternative imaging method for preoperative staging of ESCC.

## Data Availability Statement

The original contributions presented in the study are included in the article/supplementary material. Further inquiries can be directed to the corresponding authors.

## Ethics Statement

The studies involving human participants were reviewed and approved by Ethics Committee of the Peking University Cancer Hospital & Institute. The patients/participants provided their written informed consent to participate in this study.

## Author Contributions

FW, ZY, and NL contributed to the study’s conception and design. Material preparation, data collection, and analysis were performed by FW, RG, and NL. Image acquisition was performed by YZ and BY. The statistical methods were reviewed by XM, HK, and YY. The first draft of the manuscript was written by FW and was revised by NL and ZY. All authors contributed to the article and approved the submitted version.

## Funding

This study was funded by the National Natural Science Foundation (No. 81871387; No. 81871386), Beijing Natural Science Foundation (No. 7202027).

## Conflict of Interest

Authors HK and YY were employed by Beijing United Imaging Research Institute of Intelligent Imaging, UIH Group.

The remaining authors declare that the research was conducted in the absence of any commercial or financial relationships that could be construed as a potential conflict of interest.

## Publisher’s Note

All claims expressed in this article are solely those of the authors and do not necessarily represent those of their affiliated organizations, or those of the publisher, the editors and the reviewers. Any product that may be evaluated in this article, or claim that may be made by its manufacturer, is not guaranteed or endorsed by the publisher.
